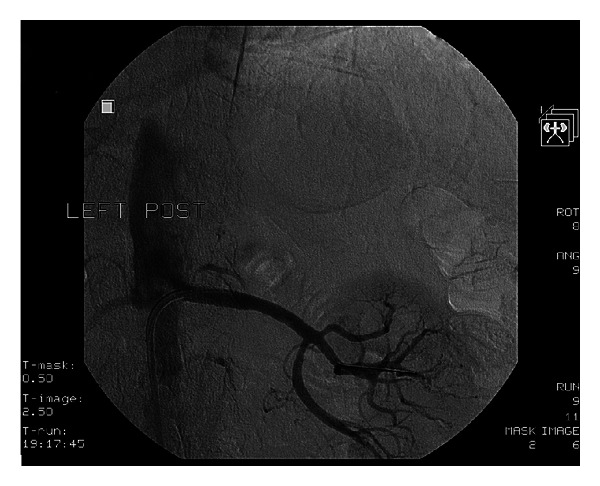# Modification of the No-Touch Technique during Renal Artery Stenting

**DOI:** 10.1155/2013/516267

**Published:** 2013-05-29

**Authors:** John A. Stathopoulos

**Affiliations:** Columbia University, 30-10 38th Street, 2nd Floor, Astoria, NY 11103, USA

## Abstract

Renal artery stenting has been established as the primary form of renal artery stenosis revascularization procedure. The no-touch technique is proposed in order to avoid renal artery injury and atheroembolism during renal artery stenting. We describe a modification of the no-touch technique by using an over-the-wire (OTW) balloon or a Quickcross 0.014′′ catheter with a 0.014′′ coronary wire inside, instead of the rigid 0.035′′ J wire. The reported technique, while it prevents direct contact of the guiding catheter with the aortic wall, at the same time it allows for a closer contact with the renal arterial ostium and a more favorable guiding catheter orientation, compared to what is achieved with the use of the more rigid 0.035′′ J wire, thus improving visualization, reducing the amount of contrast required, and potentially decreasing complications.

## 1. Introduction

 Renal artery stenting has been widely used for the treatment of renal artery stenosis. The technical aspects of stenting have improved over the last years, and procedural safety is recognized as of paramount importance. Two invasive techniques are proposed in order to avoid renal artery injury and atheroembolism during renal artery stenting [1]: the catheter-in-catheter and the so-called no-touch technique. The no-touch technique [2] uses a 0.035^″^ J wire inside the guiding catheter, to lift the tip off the aortic wall. With the 0.035^″^ wire in place, the guiding catheter is aligned with the renal artery, and a 0.014^″^ guidewire is used to cross the stenosis. The 0.035^″^ wire is then removed, and the guiding catheter is advanced over the 0.014^″^ wire to engage the renal artery.

 We report a modification of the no-touch technique by using an over-the-wire (OTW) balloon or a Quickcross 0.014^″^ catheter (Spectranetics) with a 0.014^″^ coronary wire inside, instead of the rigid 0.035^″^ J wire. 

## 2. Case 1

 A 67-year-old lady, with uncontrolled severe hypertension despite therapy, peripheral arterial disease (PAD), and left ventricular hypertrophy was diagnosed with right renal artery stenosis and referred for renal angiography.

 An abdominal aortogram confirmed the presence of significant right renal artery stenosis. Renal percutaneous transluminal angioplasty (PTA) was then undertaken. 

The procedural steps were as follows.

**Figure 1:**
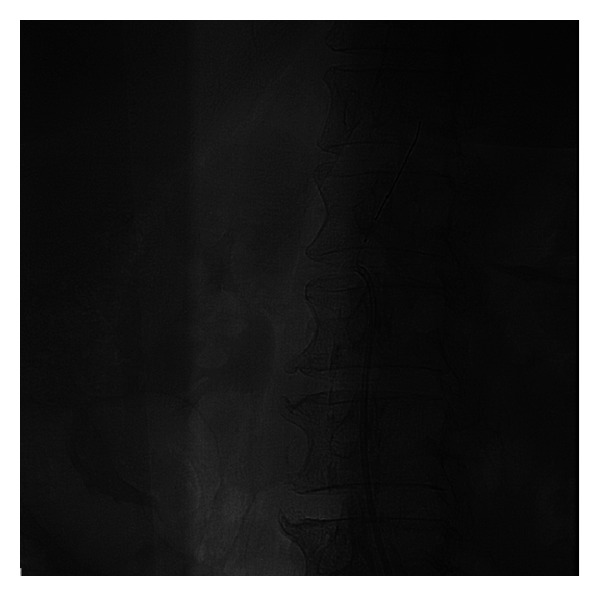


**Figure 2:**
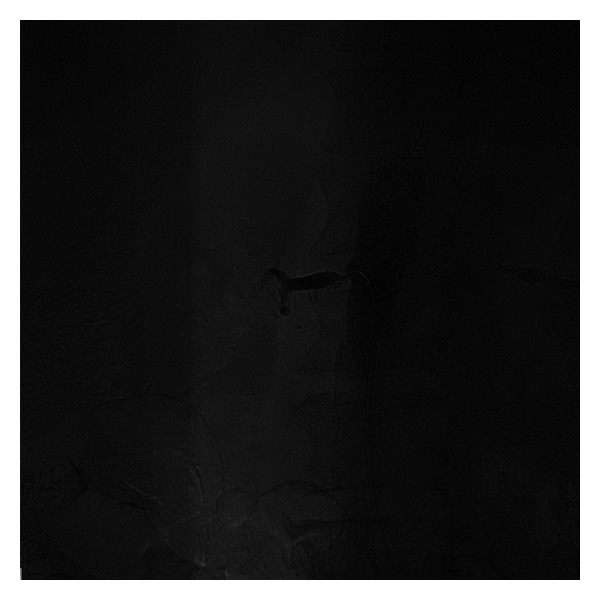


**Figure 3:**
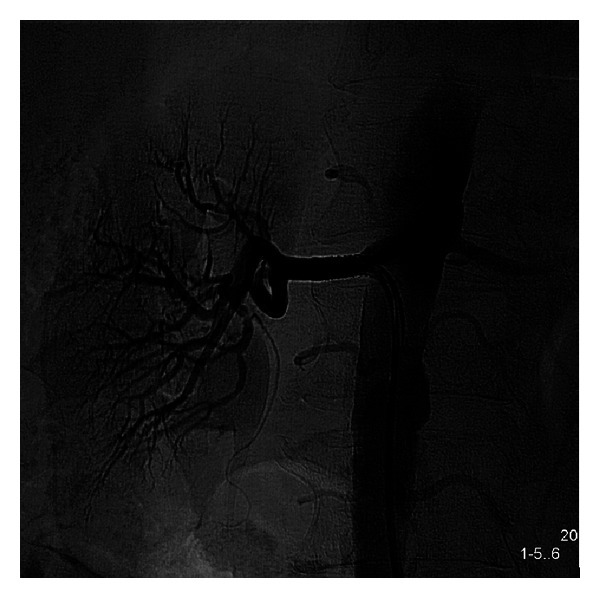


1. A 6F internal mammary artery (IMA) guiding catheter (Launcher, Medtronic) was introduced and was placed at the level of the right renal artery but pointed away of the right renal artery ostium, without touching the aortic wall.
2. A 0.014^″^ Balance (Abbott) coronary wire in a 0.014^″^ Quickcross catheter (Spectranetics) was introduced in the 6F guiding catheter with the tip of the wire protruding about one inch outside the Quickcross catheter (Spectranetics) and was advanced outside and above the tip of the guiding catheter towards the more proximal abdominal aorta (at a higher level than the ostium of the renal artery).
3. With the Balance wire (Abbott) and Quickcross catheter (Spectranetics) protruding about two inches outside the guiding catheter, the guiding catheter was manipulated and oriented towards the ostium of the right renal artery (Figure 1). The guiding catheter was cleared of blood and possible debris.
4. The ostium of the right renal artery was identified by injecting small puffs of contrast without direct contact of the angulated tip of the IMA guiding catheter with the aortic wall.
5. While in front of the ostium and despite the fact that the guiding catheter was not engaged, not touching the ostium of the artery, selective angiography of the right renal artery was performed revealing 85% stenosis (Figure 2).
6. Subsequently, first, the Balance wire (Abbott) was retracted inside the Quickcross catheter (Spectranetics), protecting the tip of the wire, and then, second, the Quickcross catheter (Spectranetics) was retracted slowly inside the IMA guiding catheter allowing for the gentle cannulation of the right renal artery ostium. In that way, scraping of the aortic plaque from the guiding catheter manipulations during renal artery ostium cannulation was minimal.
7. Then, the Balance wire (Abbott) was advanced across the lesion into the distal renal artery.
8. The Balance wire (Abbott) was exchanged through the Quickcross catheter (Spectranetics) for a Stabiliser Plus 0.014^″^ wire (Cordis), and the lesion was predilated with a 3.5 × 12 mm Trek RX balloon (Abbott).
9. A 5.0 × 15 mm Herculink Elite RX stent (Abbott) was then deployed across the lesion and flaring postdilatation performed with the stent balloon. Subsequent angiography revealed optimal stent deployment and absence of peripheral embolization, dissection, or perforation (Figure 3).

Three days after the procedure, the patient experienced generalized rash attributed to clopidogrel, and prasugrel was started instead. Repeat blood pressure at the office was only mildly elevated despite the fact that the patient had stopped taking the prescribed antihypertensive medications.

## 3. Case 2

**Figure 4:**
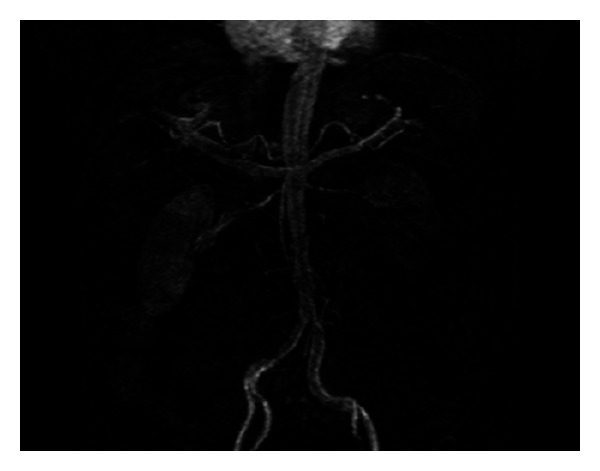


**Figure 5:**
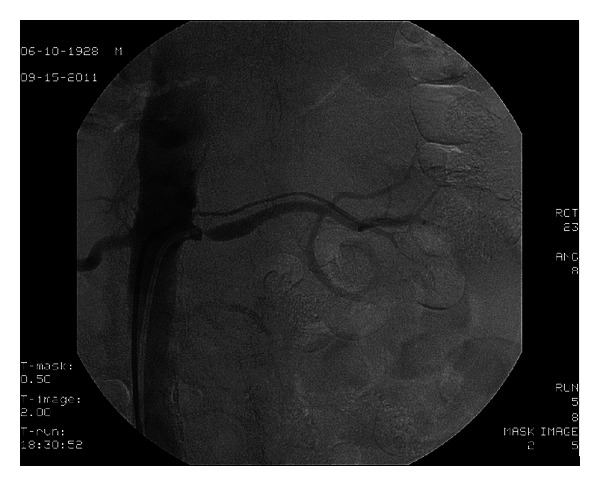


 An 83-year-old gentleman, with chronic renal insufficiency, coronary artery disease and PAD, resistant hypertension, and intolerance to angiotensin converting enzyme (ACE) inhibitors, was diagnosed with severe left renal artery stenosis by magnetic resonance angiography (MRA) (Figure 4). Selective angiography of the left renal artery was performed revealing eccentric subtotal 98% occlusion (Figure 5).

 Renal artery angioplasty was decided, and the same procedural steps were performed as described in Case 1, but instead of a 0.014^″^ Quickcross catheter (Spectranetics), we used an OTW Sprinter (Medtronic) 2.5 × 12 mm balloon.

A 6.0 × 15 mm Herculink Elite RX stent (Abbott) was deployed across the lesion. Subsequent angiography revealed optimal stent deployment and absence of complications (Figure 6). Overall, less than 15 cc of contrast was used for the renal angioplasty. Three weeks after the procedure, creatinine was stable and blood pressure was 130/90 mmHg on two antihypertensive medications (decreased from three prior to procedure).

## 4. Discussion

 Renal artery stenting has been established as the primary form of renal artery stenosis revascularization procedure. Although, the renal vascular bed is considered more forgiving than other vascular beds to potential complications during renal artery stenting, the no-touch technique [2] is proposed in order to avoid renal artery injury and atheroembolism [1]. The no-touch technique [2] uses a 0.035^″^ J wire inside the guiding catheter, to lift the tip off the aortic wall. With the 0.035^″^ wire in place, the guiding catheter is aligned with the renal artery, and a 0.014^″^ guidewire is used to cross the stenosis. The 0.035^″^ wire is then removed, and the guiding catheter is advanced over the 0.014^″^ wire to engage the renal artery.

 We describe our experience by using either an OTW balloon or a 0.014^″^ Quickcross (Spectranetics) support catheter with a 0.014^″^ coronary wire inside in order to reduce direct contact with the aortic wall. While this prevents direct contact with the aortic wall, at the same time it allows for a closer contact with the ostium and a more favorable guiding catheter orientation towards the renal artery ostium—especially with angulated oriented ostia, compared to what is achieved with the rigid 0.035^″^ J wire. This allows for a better visualization of the ostium with injection of even small contrast puffs. Furthermore, the use of the described modified no-touch technique may reduce the theoretical higher risk of aortic injury by the use of the 0.035^″^ rigid J wire.

 Alternatively, the described modified no-touch technique can be also performed with the use of a 5F guiding catheter (0.058^″^ inner diameter).

 In conclusion, the reported technique by using an OTW balloon or the Quickcross catheter (Spectranetics) with a 0.014^″^ coronary wire inside, while it prevents direct contact of the guiding catheter with the aortic wall, at the same time it allows for a closer contact with the arterial ostium and a more favorable guiding catheter orientation, compared to what is achieved with the use of the more rigid 0.035^″^ J wire, thus improving visualization, reducing the amount of contrast required, and potentially decreasing complications.

## Figures and Tables

**Figure 6:**